# Collaborating with a Graduate Marketing Class to Boost Awareness of the AIDS and Cancer Specimen Resource

**DOI:** 10.1089/bio.2021.0025

**Published:** 2021-10-18

**Authors:** Lynda M. Maddox, Sylvia Silver

**Affiliations:** ^1^ Department of Marketing, George Washington University, Washington, District of Columbia, USA.; ^2^ Department of Microbiology, Immunology and Tropical Medicine, George Washington University, Washington, District of Columbia, USA.

**Keywords:** ACSR, marketing, collaboration, social media

## Abstract

The AIDS and Cancer Specimen Resource (ACSR) was built and maintained to support research into cancers in individuals infected with the HIV by providing specimens at no charge. Numerous methods to reach investigators about this resource have been used, but the possibility of underusage of the resource has been a concern. Using a commercial marketing firm to raise awareness was not a possibility for the ACSR. However, a unique academic interdisciplinary collaboration was developed with a marketing communications course using primary and secondary marketing research and experiential learning applied to the ACSR. The cross disciplinary approach promoted greater understanding of the intersection between medical research, biobanking, and business. The collaboration resulted in new strategies to improve the visibility of the ACSR by focusing on its social media presence, creating a database of potential new users, enhancing the ACSR website, and making domain name changes. Lessons learned from this exercise should have general applicability across the field of biorepositories.

## Introduction

The AIDS and Cancer Specimen Resource (ACSR) is funded by the National Cancer Institute (NCI) to supply biospecimens and associated data to support research on cancers associated with infection with the HIV.^1,2^ The ACSR is a consortium comprising George Washington University (GW), Washington, District of Columbia; the University of California, San Francisco, California; Baylor College of Medicine, Houston, Texas; the Mayo Clinic, Scottsdale, Arizona; and Stellenbosch University, Cape Town, South Africa.^3–5^ Collectively, the consortium holds the largest collection of biospecimens and data from individuals with HIV-associated cancers in the world. These are available at no charge after an application is reviewed and deemed meritorious by an independent review panel administered by the NCI. This specialized research area is small, so requests for specimens and data are correspondingly expected to be low. In such circumstances, the possibility of underuse of the ACSR inventory is of concern.

In general, underuse of biorepository resources can occur and leads to practical and ethical problems.^6,7^ Some issues include the costs involved in maintaining unused inventories, while ethical questions are raised surrounding consenting individuals for specimens that are not used. The need to address the issue has been long recognized.^8,9^ Previous efforts have focused on past practices to identify shortcomings.^10^ The need for marketing initiatives has been identified.^11^

The ACSR tried several marketing tactics to increase knowledge of its existence and interest in its inventory, for example, using a colorful logo consistently throughout all marketing efforts, developing a website explaining the types of specimens available, exhibiting at HIV/AIDS and cancer meetings, distributing promotional items like notebooks, and sending out email blasts about ACSR operations and HIV cancers. After the ACSR distributed promotional materials at pertinent conferences, there would be an uptick in inquiries and requests for specimens, but these usually waned with time. These various efforts yielded only minimal cumulative returns as measured by the annual distribution statistics of the ACSR inventory. Although hiring a professional marketing agency was beyond the scope ACSR's budget, tapping into the resource of marketing students was within its reach. This idea was the kernel of an interdisciplinary collaboration at the GW between the School of Business and the School of Medicine and Health Sciences.

The GW Business School's Department of Marketing offers a yearly graduate marketing communications course that uses experiential learning and a “real-world” client.^12,13^ Potential projects are selected based on client need and student learning opportunity. Previous clients have included a major automobile company, a household product company, and a disinfectant product company. Although the ACSR was an unusual client compared to the more mainstream consumer goods, it provided the students with a good opportunity to learn about a field unfamiliar to them.

## Methods

A Project Brief was developed, posted on GW's online portal (Blackboard) and covered the following:

- Background of the ACSR, its mission and funding by the National Institutes of Health (NIH) NCI.
- Methods used by ACSR to build and maintain the Inventory.
- Review of ACSR data on previous requestors and users, prior marketing initiatives, and number of hits on its website following various marketing efforts.
- Geographic scope of the ACSR.
- Objective to increase the number of individuals, both domestically and globally, requesting and using specimens and data from the ACSR.
- Objective to identify and increase activity at all stages of researchers' decision processes.
- Summary of deliverables, including a 20-minute video “pitch” of ideas and live question and answer (Q&A) session to the ACSR.

The students were divided into three teams of four to six students per team. Each team was expected to independently develop a marketing plan for the ACSR “client.”

Since most of the students did not have scientific backgrounds, they were provided with a baseline level of education about HIV/AIDS so that they could appreciate the consequences and sequela from HIV infection and better understand the mission of the ACSR. An introduction to the biology of HIV, the natural history of infection, effect of the introduction and history of antiviral treatment in HIV, and statistics related to the occurrence and death of cancers associated with living with HIV were provided. As is typical, most of the students were surprised at the high prevalence of HIV infection in the United States and what living with the virus means.

After this introduction, the students in each team began working on their respective marketing plan. Meanwhile, marketing lectures covered topics such as (1) determination of client needs, (2) qualitative/quantitative marketing research methods, (3) the difference between insight and research results, (4) consumer behavior theory and the consumer “journey,” (5) media planning, and (6) how to “pitch to the client.” Throughout the semester the ACSR “client” was available to answer questions. A couple of the teams visited the GW Biorepository to better understand biobanking and the operations and functions of a biorepository.

All graduate student teams working on the project conducted primary research focused on key potential users of ACSR. This involved published researchers in AIDS/Cancer research, including primary and secondary researcher and graduate students. The teams measured aided recall (brand name recognition) as well as unaided mentioning (top of mind awareness).

At the end of the semester, four members of the ACSR Executive Committee, all well experienced in the group's previous marketing strategies, became a group of high-level client judges to select the best team marketing plan. The marketing class professor took a major part in the discussions with the judges during their deliberations, offering her extensive marketing expertise and perspective on the actual marketing effectiveness of each plan.

A score sheet/rubric was provided to the judges and to the student teams. Teams were evaluated on the following: (a) doing appropriate and innovative secondary and primary research that yielded meaningful insights, understanding the client “ask” (20%); (b) setting media, communication, marketing, and creative objectives (10%); (c) media and communication strategy, creative executions, and target market/persona/consumer “journey” (35%); (d) budget recommendation/allocation and accountability measures (15%); and (e) overall presentation quality (20%).

At this stage, the course was totally online due to the coronavirus disease (COVID) shutdown at the university, and the team presentations took place through Blackboard. Each of the three teams presented its 20-minute marketing plan to the judges; afterward, the judges asked each team about their strategies. The class was recessed, and the judges caucused to decide the “winning team.” Finally, the judges selected the team they would hire if this were a real-world project and provided each team with feedback and an evaluation of their marketing plan.

## Results

The student groups independently arrived at many concepts and suggestions; some were unique contributions and others were common to several groups. The following is a compendium of the marketing strategies from the student teams:

### Target markets

Teams performed research to learn how to expand awareness and interest in ACSR. They needed to identify the best markets to reach and specify the near versus far term goals.

Their first step was to examine the size and “consumer journey” of the current user base. If the teams could discover insights about current users and their “consumer journey,” they could make recommendations about how to improve the relationship with these users and to attract others like them.

The ACSR provided the student teams with the names of current HIV malignancy research users. ACSR had identified potential respondents who said they agreed to be interviewed. The teams then independently chose who and how many people were contacted for in-depth interviews. The students learned how these current users discovered the ACSR, how they perceived the application, approval, and delivery process for specimens/data, and how the ACSR was used to facilitate their research.

Next, they looked for potential users who shared characteristics of current users. These characteristics included research expertise and the likelihood to benefit from using the ACSR in the future. They identified not only HIV malignancy researchers as potential users but also graduate and medical students. Their starting point was a database of current and past users of ACSR that was maintained by the ACSR, including information on type of user (i.e., academic vs. other institutional users) and publications/presentations stemming from use of the ACSR biospecimens. The students augmented the database by identifying key universities with researchers who had used ACSR and by looking up published information on the researchers who were listed on articles in AIDS/cancer research. They also looked at universities that had graduate research programs in these areas. The goal of the expanded database was to identify likely immediate users as well as future users. The ultimate goal was that the database could be used to grow usage now and even expand the research field in the future. After conducting surveys and in-depth interviews, one student team provided the ACSR with a database of approximately two hundred potential users and their contact information. They presented the current “customer journey” and suggested ways to find new users.

Instead of the ACSR targeting only first or senior authors of articles related to HIV and cancer, the students suggested reaching out to co-authors, thus “touching” additional possible users. They also suggested reaching out to graduate students and postdoctoral scientists to inform the next generation of possible users.

### Market growth

Other teams concurred about the need to expand the current market. Some suggested that the ACSR needed to help new users think about research potential. By focusing only on those who were interested in AIDS malignancy research, the market would remain relatively small. If young researchers and/or influencers in either the field of HIV or cancer research saw the potential for having access to samples, it might be possible to expand the market. This would be a long-term rather than short-term goal.

### Increasing brand awareness

Every team evaluated the ACSR's brand recognition (aided recall) and awareness. This was accomplished by showing survey and in-depth interview respondents a list of real and fictional names of biospecimen banks and asking them to check those they had heard of. In this way, brand recognition was found to be nearly at zero, even among key potential users.

When searching ACSR online, a company selling aluminum-conductor steel-reinforced cable was listed before the ACSR, signaling to the teams that the ACSR's present domain name needed to be changed and interactivity increased to facilitate search engine optimization (SEO) ranking. The domain name ACSR.com was already taken, so ACSR1.com was suggested and it was available in .com and .org. This domain duplicity increases chances that the resource can be found and improves search result priority.

### Increasing social media presence

The students also stressed the importance of increasing social media presence.^14^ Although the ACSR website had logos for social marketing platforms, these were never activated. LinkedIn was identified as a key social/professional site to target. ACSR staff and researchers were encouraged to set up LinkedIn accounts. Facebook and Twitter were also suggested. The need to curate content relevant to events, research, and publications was identified. The students developed a video showing an ACSR banking site and describing the ACSR mission. Development of similar videos such as explaining how to request specimens or showing various biobanking techniques was suggested. Students recommended that these videos be posted on social media and on the ACSR website. As a result, the Marketing Committee of the ACSR has established LinkedIn, Twitter, and Facebook pages and is planning to post videos.

### ACSR website suggestions

Students found the ACSR website attractive; however, they believed that the concept regarding no charge for specimens and data did not figure prominently enough in any of the website information. All teams found that this fact should be prominently and pointedly displayed. Primary research had shown that this complimentary access for researchers was a key benefit in attracting current researchers in the field and fostering the development of new projects.

The first step teams took in doing the primary research was to conduct qualitative research (in-depth interviews) to identify key aspects of the ACSR that current and potential users found attractive or unattractive about the ACSR, its services, and its current marketing activities. They then developed survey instruments that allowed them to quantify the importance of factors gleaned from the in-depth interviews. The fact that specimens were provided to researchers free of charge was rated highly.

The application and fulfillment processes were seen as lengthy and cumbersome by current users. Once customers landed on the ACSR website, they were required to click multiple times before arriving on the page for requesting specimens. Long lag times and seemingly endless bureaucracy (such as requiring sign-off by an institutional official ensuring that the applicant had funding to perform the stated research question or having an *ad hoc* review of the science and statistics underlying their request) caused some investigators to abandon their proposals to request specimens.

### Conference attendance

Students found that the ACSR had relied predominantly on in-person meetings/conferences attended by those in HIV malignancy research. Although a person-to-person contact is often viewed as the highest quality interface, ACSR members did not have ACSR business cards with contact information. In addition to personal contact, ACSR provided notebooks and other items with its logo. However, many of the logo items lacked contact information; so potential customers could not easily contact someone with further interest.

In addition, as more conferences moved online due to COVID-19, there was a greater need for seamless contact, database building, an easy domain name, and quick follow-up. Even after the pandemic, there will likely be more virtual conferences increasing the need for ACSR to develop and increase its online presence through a better domain name, SEO, use of social media, better processes, and a more succinct message to potential users.

### Development of leadership and education

The ACSR, despite its decades of experience in biobanking, did not present any continuing education offering. Student teams suggested that ACSR focus and highlight research advancements made through use of ACSR-provided specimens.

Currently, those receiving specimens from ACSR are “required” to acknowledge the ACSR in resulting publications. Oftentimes, this does not occur, either because early discovery research does not result in publications or investigators inadvertently forget to acknowledge the ACSR in their articles or meeting presentations. Acknowledgment should be standardized and enforced, and ACSR should make it beneficial for researchers to do so. An important part of the marketing plan is the dissemination of research publications containing ACSR acknowledgment to key opinion leaders and influencers in the research community. As key top researchers identify and use the ACSR, the more it strengthens brand recognition and potentially increases usage.

### ACSR implementation

The Marketing Working Group of the ACSR has begun implementing some of the recommended marketing strategies with planned evaluation metrics. Some ideas could be implemented rapidly, while others will take more planning and exploration. Evaluation matrices include activity on social media sites; for example, how many “likes” the ACSR receives on Facebook, how many ACSR connections appear on LinkedIn, and comments/messages mentioning the ACSR. Of course, the most advantageous outcome of this initiative would be an increase in requests for specimens and data, which will need to be determined.

The ACSR adopted two new domain names, ACSR1.com and ACSR1.org. Both bring the user to the same ASCR webpage. The domain name is also carried on all ACSR products addressing the need for consistency across strategic products.^15^ Changes made to the ACSR website include prominently displaying its mission that it is a source of HIV-associated malignancy specimens to researchers *at no charge*. The emphasis on “no charge” helps allay the perception of “excess bureaucracy” since the reward for the effort in requesting specimens or data seems more obtainable. In addition, a direct path now links the user from the first page of the website to the application for samples, and therefore fewer clicks. Email announcements now include additional targeted individuals using a database suggested by the class.

The student teams demonstrated that a large majority of medical science researchers are on social media; therefore, the ACSR has developed a presence on Facebook, Twitter, and LinkedIn. Also, ACSR members have linked the ACSR to their social medial accounts. As a result of these activities, the ACSR Brand is being recognized and expanded through linkages to individuals and organizations related to biobanking, which expands awareness to the ACSR and its mission.

The ACSR is beginning to introduce initiatives to better connect through online conferences and to build on the researcher database that the students started. Over the long term, the database will become a key tool in establishing and fostering relationships. These are expected to increase usage of the ACSR among current users, reach existing AIDS malignancy researchers, and even expand the field of researchers in the area.

## Discussion

Although ACSR personnel had taken on the “marketing responsibilities,” they did not have a background in marketing. Collaborating with a graduate marketing class produced a deep evaluation of the ACSR's prior marketing campaigns. In addition, a marketing program from the world of advertising evolved, a sphere out of the realm of most of the ACSR members. The ACSR can now use a “professionally defined marketing plan” based on primary and secondary marketing research, a commodity that would not be within its financial ability. The realm of HIV-associated malignancy research is a small niche in the huge area of HIV research. The marketing attempts ACSR puts forth from this activity may eventually produce more HIV malignancy researchers; additionally, the strategies to inform the researchers of the availability of a rich resource of specimens and data should be better situated to reach them.

The Business School students expanded their horizons. They realized the importance of marketing in a field they had not thought about before, and they were able to help a client at the “grassroots level.” They benefitted from the philosophy of experiential learning,^12,13^ which offered them a real-world situation to delve into an unknown “product” and to produce meaningful, effective marketing strategies.

The ACSR has already experienced an increase in activity surrounding the initiatives implemented as a result of this collaboration. The website has seen about a 10% increase in viewers compared to a comparable period of time before the change of its domain name. Also, the ACSR now has followers on Twitter, likes on Facebook, and connections on LinkedIn. These connections allow the ACSR to engage with its community of interested researchers and to post direct news updates about the ACSR, new biospecimen science results, funding availabilities from NIH, as well as the ACSR's Young Investigator Pilot Award program details. Being connected on social media keeps the ACSR actively “present” instead of just having a transient awareness that accompanies sporadic strategic marketing attempts.

Both the ACSR and the Business School Marketing Class gained much from this university partnership. The project promoted cross disciplinary collegiality and it represents a way forward for greater understanding of the intersection between medical research, biobanking, and business.

Other biorepositories like the ACSR are most likely also composed of individuals focused on science and not marketing. These entities can take advantage of what was learned through this exercise and adapt our findings according to their missions. Key points are as follows: (1) facilitate the potential user's journey in finding the biorepository online; (2) pay attention to SEO and biorepository domain name; (3) recognize that many individuals are impatient causing them to abandon an internet search after more than two “clicks”; (4) establish a social media presence and use it to link potential users to the biorepository website; (5) develop a database of relevant researchers that are users and potential users of the biorepository; and (6) actively engage researchers through contacts made at conferences. These approaches should have general applicability across the field of biorepositories.
